# Special Issue “Mechanism of Enzyme Catalysis: When Structure Meets Function”

**DOI:** 10.3390/ijms27041887

**Published:** 2026-02-16

**Authors:** Ivana Leščić Ašler

**Affiliations:** Laboratory for Chemical and Biological Crystallography, Division of Physical Chemistry, Rudjer Boskovic Institute, Bijenicka cesta 54, 10002 Zagreb, Croatia; ivana.lescic.asler@irb.hr; Tel.: +385-1-4561111; Fax: +385-1-4680245

While genome DNA can be called “a blueprint for life”, the realization of this blueprint is the responsibility of proteins [1]. They are the main catalysts, structure components, signal transfers, and molecular machines in a biological organism [1]. It is generally accepted that the gene sequence determines the amino acid sequence, the amino acid sequence determines the protein structure, and the protein structure determines its function. Advances in sequencing technologies and computational power have provided a large increase in sequence information—currently (December 2025), there are over 250 million nucleotide sequences in the GenBank [2] database. However, only ~570,000 protein sequences are listed in SwissProt, the manually annotated and reviewed portion of UniProtKB [3], and only a small percentage of these have experimentally determined functions. On the other hand, there are almost 90,000 non-redundant protein sequence 3D-structures in the Protein DataBank [4], and many of them are not annotated (e.g., some of the ~16,500 structures from structural genomics projects). Many bioinformatic tools have been developed to predict the protein function from its sequence/structure, but they have so far been mainly based on transferring the relatively small number of experimentally determined functions to large collections of proteins based on sequence similarity, which still poses significant challenges [5]. This practice of assigning function based on sequence or structure similarity has caused mis-annotations in databases. It is estimated that sequence identities of 60% or higher will transfer function incorrectly in 10% of cases, and the mis-annotation level is even up to 63% across six superfamilies, according to [6].

There is no doubt that the function of proteins and, more specifically, enzymes is intimately linked with their three-dimensional structure. The enzyme size, shape, and charge of the active site, interactions between domains and/or subunits, protein dynamics, existence of cofactor and/or allosteric sites, conservation of catalytic residues are just some of the key structural features that reveal the mechanistic details of molecular function and lead to a hypothesis about how a given enzyme operates [7]. Ribeiro et al. [8] sorted enzyme properties into six dimensions that need to be studied and thus obtained knowledge integrated in order to fully understand a particular enzyme: sequence, structure, ligand binding, catalytic site, catalytic mechanism, and reaction catalyzed. With the development of the artificial intelligence (AI) tool Alpha Fold for the prediction of a protein’s 3D structure, a breakthrough occurred enabling bridging the gap between the numbers of known enzyme sequences, available 3D structures, and functionally characterized enzymes [9]. This achievement was awarded The Nobel Prize in Chemistry in 2024 [10]. In addition, the implementation of machine-learning (ML) methodologies has significantly advanced predictive biocatalysis, providing innovative approaches to the prediction of enzyme function and the optimization of biocatalysts [11].

However, enzyme catalysis can be finely tuned by e.g., minute differences in active site or allosteric site residues, enzymes can be promiscuous, one type of reaction can be catalyzed by several enzymes, enzymes can have similar 3D-structure but low sequence similarity, and finally, AI and ML techniques are only as good as the data on which they were trained [12], and there are a lot of proteins that are not amenable to structure solving or prediction and whose function cannot be easily inferred. Therefore, in order to characterize an enzyme sufficiently, so it can be used, e.g., as a target for drug design or as a biocatalyst for industrial process, its structure and function have to be investigated in detail, using a multidisciplinary approach, collecting the knowledge from all possible sides, thus making old-fashioned biochemistry and functional studies far from redundant [7]. 

Following this line of thought, this Special Issue has collected 10 original research articles that investigate the function and mechanism of action of different enzymes via the combination of computational and experimental approaches, namely: L-asparaginase from *T. sibiricus* (Dumina et al.), bovine carboxypeptidase A (Amador Balderas et al.), human choline–acetyltransferase (Dante et al.), cytochrome TorC from *E. coli* (Panwar et al.), calf and *E. coli* purine nucleoside phosphorylase (Stachelska-Wierzchowska et al.), adenylosuccinate synthetase from *H. pylori* (Mišković et al.), *A. bisporus* tyrosinase (Montenegro et al.), *E. coli* nitroreductase (Sharrock et al.), zebrafish histone deacetylase 6 (Cellupica et al.), and haloacid dehalogenase-like superfamily phosphatase from *S. aureus* (Bang et al.). Four review articles provide an overview of the development of the analytical method of affinity electrophoresis (Masson and Pashirova), the current state of knowledge on the HtrA family proteins (Zarzecka and Skorko-Glonek), the applications of the organophosphorous degrading enzymes (Pashirova et al.), and the functional properties in the foundations of the wide applicability of 4-hydroxyphenylacetate 3-hydroxylase (Sun et al.). As Guest Editor of the Special Issue “Mechanism of Enzyme Catalysis: When Structure Meets Function”, I would like to extend my gratitude to all authors whose valuable work was published, as well as to the peer reviewers and editorial team, who contributed to the success of the edition.
